# Synthesis, Principles, and Properties of Magnetite Nanoparticles for In Vivo Imaging Applications—A Review

**DOI:** 10.3390/pharmaceutics11110601

**Published:** 2019-11-12

**Authors:** Justine Wallyn, Nicolas Anton, Thierry F. Vandamme

**Affiliations:** Université de Strasbourg, CNRS, CAMB UMR 7199, F-67000 Strasbourg, France; justine.wallyn@ics-cnrs.unistra.fr

**Keywords:** magnetite, nanoparticles, superparamagnetism, syntheses, characterizations, in vivo imaging

## Abstract

The current nanotechnology era is marked by the emergence of various magnetic inorganic nanometer-sized colloidal particles. These have been extensively applied and hold an immense potential in biomedical applications including, for example, cancer therapy, drug nanocarriers (NCs), or in targeted delivery systems and diagnosis involving two guided-nanoparticles (NPs) as nanoprobes and contrast agents. Considerable efforts have been devoted to designing iron oxide NPs (IONPs) due to their superparamagnetic (SPM) behavior (SPM IONPs or SPIONs) and their large surface-to-volume area allowing more biocompatibility, stealth, and easy bonding to natural biomolecules thanks to grafted ligands, selective-site moieties, and/or organic and inorganic corona shells. Such nanomagnets with adjustable architecture have been the topic of significant progresses since modular designs enable SPIONs to carry out several functions simultaneously such as local drug delivery with real-time monitoring and imaging of the targeted area. Syntheses of SPIONs and adjustments of their physical and chemical properties have been achieved and paved novel routes for a safe use of those tailored magnetic ferrous nanomaterials. Herein we will emphasis a basic notion about NPs magnetism in order to have a better understanding of SPION assets for biomedical applications, then we mainly focus on magnetite iron oxide owing to its outstanding magnetic properties. The general methods of preparation and typical characteristics of magnetite are reviewed, as well as the major biomedical applications of magnetite.

## 1. Introduction to Magnetism: From Particles to Nanoparticles

Currently, it is well known that some particles and nanoparticles (NPs) exhibit magnetic properties. As a matter of fact, magnetism properties are not only related to the composition of nanomaterials but are also size-dependent. Thereby, particles and NPs possess their own magnetism due to their size and will not behave similarly under a magnetic field. To explain why there are differences in behavior depends on the size of magnetic materials, the microstructure of particles, and first, we briefly introduce NPs.

**Figure 1:**
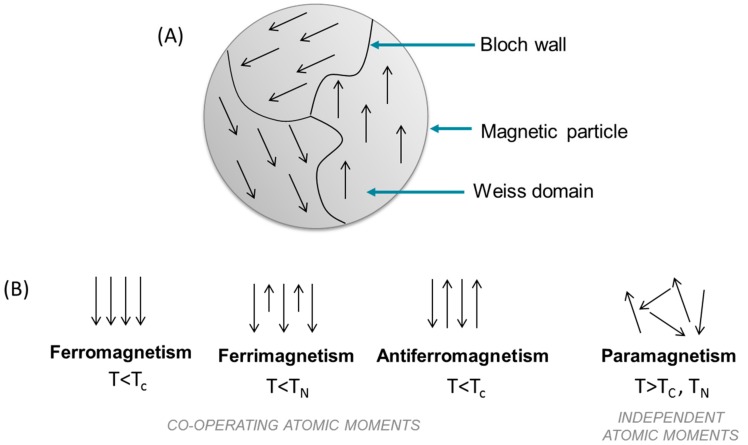
(**A**) Schematic representation of the internal structure of a ferromagnetic particle showing Weiss domains separated by Bloch walls. (**B**) Types of magnetism (Reproduced with permission from Amyn S. Teja et al., Progress in Crystal Growth and Characterization of Materials; published by Elsevier Ltd., 2009. from ref [5]).

In magnetic applications, particles with an average diameter up to 100 nm are generally called bulk material; whereas below this size, particles belong to the category of NPs. In the first case, bulks made of ferromagnetic, ferrimagnetic, or antiferromagnetic particles possess a microstructure composed of many magnetic domains called Weiss domains separated by walls named Bloch walls as shown in Figure 1A. Those domain walls aim to reduce magnetic stray energy. In every domain of the particle, a collective coupling of spins makes spins is collinear along one direction due to magnetic anisotropy energy [1,2,3].

On the contrary, bulk is made of multi magnetic domains, typically inferior to 100 nm, where the magnetization is uniform in any of them but different from one domain to another one. At room temperature and under a zero magnetic field, all domains have their own spin alignment rendering the bulk material macroscopically non-magnetic or with a global magnetization near zero due to the balance of every Weiss domains’ magnetism by their next neighbor domains (Figure 1A). However, an external magnetic field can induce wall movement—or in other words domain nucleation—in the case of a small magnetic field or, in the case of large magnetic field, make every spin from each single Weiss domain rotate away from their easy axis magnetization and be reoriented along an unique and same direction aiming to turn the bulk material into a magnet. This uniform and finite magnetization persists as long as the magnetic field is applied and stops right after its removal [2,3,4]. Temperature (T) has also an influence on magnetic particles behavior (Figure 1B). As previously mentioned, at room temperature and under a zero magnetic field, magnetic moments interact to align themselves in every magnetic domain. The influence of the interaction, called exchange interaction, can be offset by reaching Curie temperature (TC) or Néel temperature (TN), respectively for ferrimagnetic and ferromagnetic particles and for antiferromagnetic particles [5] causing spin disorder in every Weiss domain. At Curie and Néel temperatures, all single magnetic domains contain disoriented spins and cannot be then distinguished from each other. The bulk material seems at that characteristic temperature to be composed of one big single disordered domain which means a paramagnet (paramagnetism state) where the global magnetization is also balanced and near zero [2].

Since magnetism is size-dependent, the particle size has to reach a low diameter, the so-called critical small diameter (D), to consider a particle as a single-domain particle, i.e., a NP. The limit diameter is equal to:

(1) $$D=\frac{18\sqrt{A\cdot K_{eff}}}{\mu_{0}\cdot M_{S}^{2}},$$

where *A* is the exchange constant, *K_eff_* is the effective anisotropy constant, *M_s_* is the magnetic saturation, and *µ_0_* is the vacuum permeability [1,4].

NPs or single domain particles are generally smaller than 100 nm and saturated even in the absence of an external magnetic field in reason of their size comparable to a magnetic domain size. Above a specific temperature called the blocking temperature, NPs exhibit a superparamagnetic (SPM) behavior, like particles exhibiting paramagnetic behavior above the Curie or Néel temperature. Such a change of the magnetic behavior is due to a spin reversal, contrary to particles for which temperature causes spins disorder. A spin relaxation time (*τ*), corresponding to the time needed for spin reversal, is attributed to this phenomenon and is expressed by the Arrhenius formula from Néel-Brown theory [1,2,4,6]:

(2) $$\tau =\tau_{0}\cdot exp\left(\frac{K_{eff}\cdot V}{k_{B}\cdot T}\right),$$

where *τ_0_* is the expected time of spin reversal (order of 10^−9^ to 10^−12^), *K_eff_* is the anisotropy constant of the material, *V* is the NP volume, *k_B_* is the Boltzmann constant, and *T* is the temperature.

It should be pointed out that magnetic behavior of NPs has to be described, like for magnetic bulk, for well-defined conditions of the experiment like temperature, magnetic field intensity, and experimental time scale. If *τ* is shorter than the experimental time scale (t), SPM property will be notified since magnetic moments will be reversed so many times that the magnetization of the NP will seem close to zero since all magnetization measured would cancel each other by summation. On the contrary, if *τ* is longer than the measure time, NPs’ magnetism will be described in a blocked-state (and so characterized as ferromagnetic, ferrimagnetic or antiferromagnetic depends on the magnetic ordering “observed”) since magnetic moments do not have enough time to rotate.

In many experiments, measurement time is constant but temperature is increased inducing a transition to SPM state. This temperature of transition, aforesaid under the name “blocking temperature”, is thereby described as the temperature separating two magnetic regimes. To pass from blocked-state regime to SPM regime, the energy barrier (*K_eff_·V*) has to be overcome or at least balanced by the thermal energy (*k_B_·T*) (*k_B_·T* > *K_eff_·V*). Thus, the smaller the NP is, the easier thermal energy exceeds the energy barrier at a low temperature. At this point, NP acts as a giant magnetic moment, generally called superparamagnet. That is why temperature can also induce state transition, since above the blocking temperature, the thermal energy is high enough to reverse spins, which causes a decrease of magnetization so that the transition occurs from blocked-state to SPM regime. Such a change caused by thermal conditions is approximately similar to amagnetic particle becoming a paramagnet, but in the precise case of a nanoscale magnet, a NP becomes a superparamagnet thanks to a collective movement of spins and not a fuzzy spins disorder as commonly occurs in particles [1,2,4,6].

**Figure 2:**
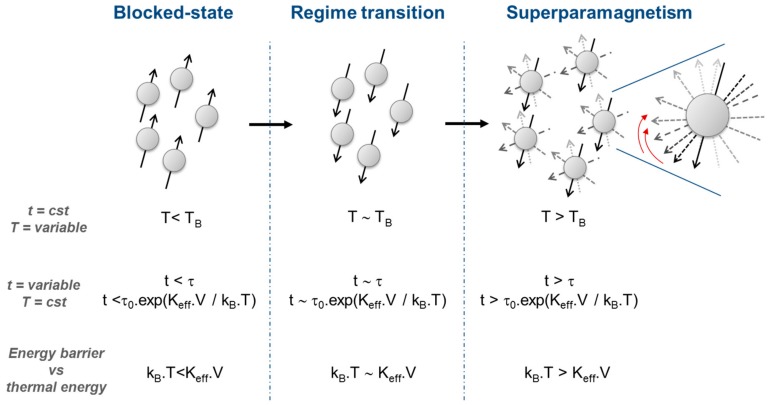
Magnetic state transition from blocked-state to superparamagnetism of a single-domain nanoparticle (NP) depending on time of measurement and temperature conditions under zero magnetic field.

Figure 2 displays a schematic magnetic regime transition from blocked-state to superparamagnetism along with variation of temperature or impact of measuring scale t.

**Figure 3:**
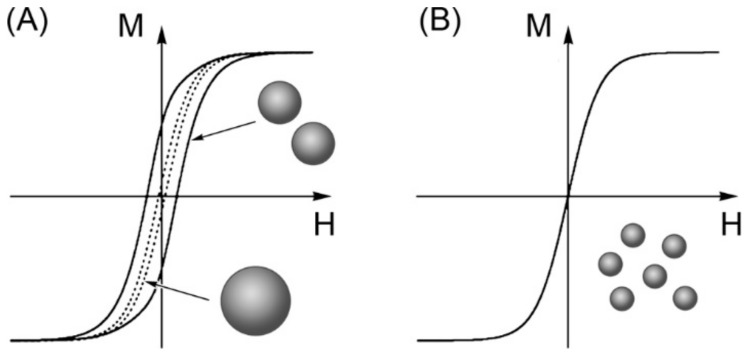
Magnetic responses of (**A**) ferromagnetic particles with a range of sizes from micron scale (multi-domain) to nanometric scale (single-domain particles) and (**B**) superparamagnetic (SPM) NPs (single-domain).

Differences between bulks and NPs can be extended to other magnetic notions like coercivity. The latter magnetic term is basically defined as the intensity of the magnetic field required to reduce magnetization to zero. Generally spin reversal for NP transition from blocked-state to SPM regimes requires more energy than domain nucleation for the magnetic transition of bulk material. Therefore getting NPs magnetism back to a stable magnetic regime, referring to the blocked-state, implies higher coercivity than particles needed for its own transition back to a stable magnetic state [2]. To keep bringing to light some nanomagnetism notions, a rigorous approach will then present hysteresis loops. This second magnetic notion is currently qualified as the magnetization cycle or the magnetic response of magnetic materials under a magnetic field. A large amount of information about magnetic properties of a material can be learned by studying its hysteresis cycle. It is mostly described by monitoring the variation of degree of magnetization (B) with the intensity of magnetic field (H), commonly referred as B–H loop. Such a curve is obtained thanks to the “memory” of the magnetic material. In other words, magnetic particles can retain the memory of an applied field once it is removed. The shape of loops is size-dependent. The presence of several Weiss domains in particles induces hysteresis as all magnetic domains do not return to their original magnetization, because they all respond to a force. The magnetic field depends on their own history with that force. For particles small enough to be single domain but not small enough to be NPs, there are broader hysteresis loops because more energy is required to make spin reversal than walls move occurring in multi-domain particles under a magnetic field (Figure 3A). In case of NPs, there is an antihysteretic B–H curve in a sigmoidal curve fashion (Figure 3B) [6].

**Figure 4:**
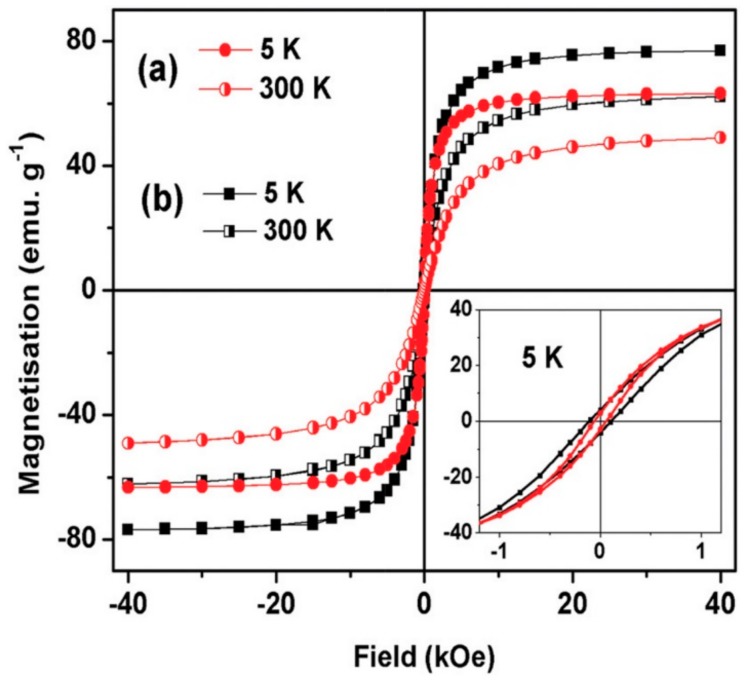
Example of hysteresis loops of (a) 4.3 nm and (b) 6.0 nm magnetite NPs at 5 K and 300 K (Reproduced with permission from Abdulwahab, K et al., Dalton Transactions; published by Royal Society of Chemistry, 2013. from ref [7]).

Once more, the temperature can have a significant impact on material magnetism and be observed on hysteresis loops. An example of typical NP hysteresis loops is shown in Figure 4 representing two trials of hysteresis cycle of 4.3 nm and 6.0 nm magnetite SPM (iron oxide NPs (IONPs)) (SPIONs) at 5 K and 300 K. Thanks to those curves, the dependence of hysteresis loops to the size of a magnetic material and the temperature can be clearly highlighted.

**Figure 5:**
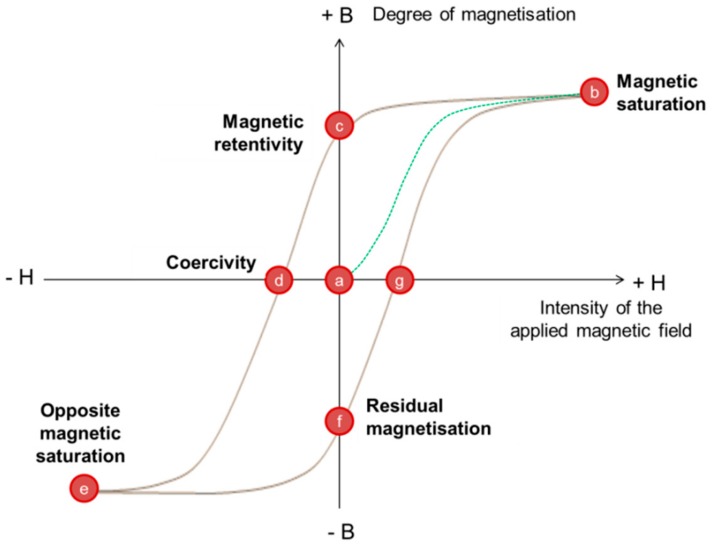
Typical hysteresis loop of a magnetic material.

The main characteristics of hysteresis loops can be pointed out in Figure 5. In Figure 5, starting from a zero magnetic field (point “a”) and then applying a high external magnetic force (H), magnetization of magnetic material reaches a maximum called the magnetic saturation (point “b”) and levels off. The material is magnetically saturated because all spins are aligned. When H is reduced to zero (from “b” to “c”) and then increased in the negative direction (from “c” to “d”), magnetization drops to zero at the point “d”. At the point “c”, the degree of magnetization B is higher than zero since the material has been disturbed by the magnetizing force and keeps a memory of it. This latter point materializes the magnetic retentivity of the material corresponding to the magnetic flux remaining within the material when H has been reduced to zero. The coercivity (point “d”) corresponds to the force needed to remove the magnetic remanence, which means reducing B to zero or, like shown on the graph from “c” to “d”. To reach the coercivity (point “d”), the reversed magnetizing force has to flip enough to reduce the degree of magnetization close to zero. If the magnetizing force H keeps decreasing in the negative direction, the material will be once more saturated but with an opposite magnetic saturation (point “e”). From “e” to “b”, H is increased back in a positive direction until the magnetization of the material moves up again to the positive magnetic saturation and makes the material complete its hysteresis cycle. However, the curve will take a different way and will not cross the point “a” or the origin of the graph to reach “b” again, owing to a residual magnetization in the material (point “f”). This is referred again to a residual magnetism in the material. Consequently, to go from “f” to “b”, B will firstly reach point “g”, strictly opposed to the coercivity point, before filling its hysteresis loop in point “b”.

Magnetic NPs currently attract scientists owing to their fascinating properties and magnetic tunability. The first reported work using magnetic NPs was published during the 1970s and was about in vitro magnetic separation of enzymes by Robinson et al. [8]. Over the next decades, applications have been extended to in vivo. As the hereinafter section of this work will show, it has already been reported that magnetic nanometer materials are quite useful for in vivo applications as nanocarriers (NCs) and nanoprobes [9,10]. NCs and nanoprobes, so-called nanoplatforms, are the design of liposomes, polymeric NPs, micelles, and inorganic materials of 1–100 nm in diameter corresponding to delivery systems and/or targeted systems. In the case of magnetic nanomaterials, this growing interest is not only due to their magnetic properties but also in reason to their nanosize providing thus a high surface-to-volume ratio, and consequently an increase in chemical reactivity, and a quantum confinement effects exerting size-dependent properties like magnetism [11,12].

## 2. Nanoscale Magnetic Material for Biomedical Applications: Current Trends and Requirements

Over the past few decades, there have been important developments regarding magnetic NPs in biomedical fields, like for diagnosis and therapy. The general term magnetic NPs takes into account structures composed of different metallic elements (iron, cobalt, nickel, platinum…), or bimetallic and metal alloys NPs. Iron may be classified separately because of its use in biomedicine as iron NPs and as iron oxide or bimetallic SPIONs. SPIONs currently play an important role in nanomedicine fields like in magnetic resonance imaging (MRI) for non-invasive diagnostic purpose. Owing to the combination of magnetism properties with nanometer scale, magnetic NPs compose a very attractive material for biomedical and technological applications [13]. They play an important role in healthcare needs due to their ability to function at the cellular scale. Due to a small controllable size and shape, magnetic NPs are comparable to biological entities and are able to easily interact with those entities or to “get close” to them [6]. Indeed, the range of sizes of NPs can be near the sizes of viruses (20–450 nm), proteins (5–50 nm), and cells (10–100 µm), depending on the methods of preparations and intended promotion of cellular uptake [6,14].

In this way, magnetic NPs have already been involved into various biomedical applications including dental applications [15], and illnesses local treatment like neurological diseases [16] and cardiovascular diseases [13]. As many reviews reported [2,6,12,17,18,19], magnetic nanomaterials are also applied for magnetic separation of cells labelled by biological entities, such as proteins [20], in bio-imaging as an MRI contrast agent, since being exposed to a magnetic field has not been contraindicated for living beings (except for patients bearing magnetizable medical devices) [21], for hyperthermia cancer therapy [22], enzyme-like mimicking SPIONs used as catalytic nanomaterials (as a new method of synthesis of SPIONs without thermal decomposition and high temperature) [23] and targeted drug or gene releases [24]. Regarding targeting strategies, it should be mentioned that the interest of local treatments rely on the fact that targeting a specific site via NP-based biomarkers [25] (such as a group of tumor cells) can reduce side effects and avoid the attack of regular cells for the treatment, and even more, the dosage can also be cut down since the cytotoxic treatment will not be uptaken by healthy cells [13,22]. It must be mentioned that most applications, and even those introduced here, require metallic ferrous NPs, like SPIONs. Investigations about magnetic ferrous NPs, mostly magnetite and maghemite, have been achieved since they have been assimilated to promising candidates for biological applications [26]. This kind of ferrous metallic NP will be thoroughly introduced in the next parts of this study.

Although magnetic NPs seem quite useful for plenty of in vivo applications, the recognition by the immune system and an eventual unfavorable biodistribution constitute their main disadvantages. First of all, they are composed of elements different from those composing natural organic biomolecules, which provokes a spontaneous immune system response. Consequently, the loose of their stealth properties leads to a fast excretion from the blood stream, a decrease of stability in physiological media, and also a risk of toxicity on the patient due to metabolites. It should be pointed out that involving magnetic NPs in therapeutic treatments requires knowing the half-life time in plasma and final biodistribution of NPs and their metabolites for an efficient and safe use [21]. Second of all, the nanometer size promotes their penetration through tissues or capillaries and sometimes causes extravasation issues after intravenous injection. Extravasation issues are quite frequent in cancerous tissues because tumor tissues possess a vascular system with a heterogeneous morphology. Current cancer treatments are based on NPs innovation offering real-time imaging and local drug release (NCs and nanoprobes). Magnetic NPs used as drug NCs have been qualified as “theranostic” or “nanotheranostic”, meaning therapeutic and diagnostic with the possibility to encapsulate guest bioactive compounds, to protect them and to gradually release them to targeted sites by guided-NPs. So, in the case of theranostic magnetic NPs for cancer treatment, extravasation issues in tumor tissue may occur even more easily than in regular tissues and may generate a decrease of the theranostic nanomaterial rate within the diseased area [2,27,28,29]. To know the fate of magnetic materials in vivo, a thorough understanding of pharmacokinetic mechanisms related to NPs is required. Essentially, the mechanism of distribution–metabolization–excretion after the exposure corresponds to a renal clearance process (filtration) of the invading NPs from the blood compartment. In the case of hydrosoluble and small NPs, the excretion will be the next step whereas lipophilic compounds and/or bigger NPs will be metabolized by others organs due to their ability to bypass renal filtration [13]. A sequestration in organs or tissues belonging to the reticuloendothelial system (RES) (like the liver, the spleen, the bone marrow, or lymph nodes) where the biotransformation mechanism occurs (opsonization, phagocytosis, and endocytosis) is so far necessary to achieve hydrosoluble metabolites which will then be excreted like other water-soluble substances. The typical biodistribution a few minutes after a NP blood stream clean up is 90% in the liver, 2% in the spleen and 8% in the bone marrow [21], but that remains highly dependent on the NP size [29]. Many other factors affect the behavior in physiological media and the biodistribution of NPs hass to be managed to prevent fast excretion of NPs and to ensure distribution to targeted zones. Those factors are mostly, as aforementioned, the size, the composition, the morphology, and the surface chemistry (charge, moieties, shells…) but recent achievements allow us to obtain magnetic NPs possessing a tunable surface which makes them inconspicuous regarding the immune system and be associated with an effective and non-toxic biomedical magnetic NP platform [13].

To obtain nanomagnets for biological uses, researchers have focused their efforts on the functionalization of magnetic NP surfaces to render them stealthy and stable in physiological media. It has been reported that NPs can be functionalized on their surfaces by lots of chemical surface modifications involving some biocompatible molecules like polymers, hydrophilic molecules, or silica layers for example [6,29]. Working on magnetic NP surface charges has also been studied in order to facilitate the interaction or adherence of those nanoprobes onto cellular membranes, which are generally negatively charged [14]. In this way, NPs can be coated to form core-shell nanostructures, functionalized or embedded in a matrix for targeting applications and with stealth properties. Such strategies could mislead the immune system, postpone the metabolism stage, and so far enhance magnetic NPs retention within the blood stream which constitutes an asset for all theranostic applications.

Therefore, the design of magnetic nanocrystals for biomedical applications needs to be well controlled in order to get specific properties dictating magnetic NPs’ fate in vivo. Otherwise an injection of magnetic NPs for biomedical purposes will be useless if their blood circulation time is not extended enough to let NPs do what they are injected for. In addition, fundamental magnetic properties have to be clear and optimized to be controlled depending on the conditions of the application [30]. In other words, synthesis of nanoscale magnets coated by organic or inorganic shells, bearing ligands capable of bonding to specific receptors, and having a specific narrow range of size and a high degree of biocompatibility has to be fully mastered [21,29]. Magnetic NPs should also exhibit high uptake efficiency in the case of targeting strategy, so as not to be subject to aggregation to prevent from vessel embolism or get structural damage after being exposed to the in vivo environment [31,32]. Even if this review is mainly focused on magnetic NPs, it has to be precise that other NPs, like polymers, liposomes, ceramics, dendrimers, and many others, have also to fulfil those specific requirements prior to envisage in vivo translation [25].

## 3. Magnetic Iron Oxide Nanoparticles

### 3.1. Three Main Iron Oxide Nanoparticles

As has already been reported in this dissertation, metallic ferrous NPs belong to the kind of NPs that are most widely applied in therapeutic, diagnosis, and real-time imaging domains. Metallic ferrous NPs referred in literature are generally based on three main iron oxides. Basically those are the hematite (α-Fe_2_O_3_), the maghemite (γ-Fe_2_O_3_) and the magnetite (Fe_3_O_4_ or FeO.Fe_2_O_3_); they are the most common in nature and mainly found in soil and rocks from volcanic eruptions and also from air pollution (emissions from traffic and industries) [5,33,34].

Categories related to their size allow us to classify them from micrometer-sized (300 nm–3.5 µm) to standard (10–150 nm) and to ultra-small (<10 nm) iron oxide crystals. Such a classification is useful to identify general advantages and disadvantages that we would have to deal with if we are using SPIONs. For example, for in vivo purposes, NPs biodistribution is size-dependent and NPs must have an optimal average diameter to avoid sequestration (occurring with micrometer-sized iron oxides) or an early renal clearance (occurring with ultra-small iron oxides) which correspond to the diameter of NPs belonging to the second class, standard iron oxide nanocrystals [11,13,25,34]. Those should be able to remain within the blood stream over an extended period of time to promote local accumulation and avoid vessel embolizations. Owing to a specific biodistribution for each category of iron oxide nanocrystals, in vivo applications are size-dependent. For example, standard and ultra-small SPIONs have respectively been used as contrast agents for the diagnostics of liver diseases due to the easy uptake or passive targeting of liver cells (Kupffer cells) and for blood-pool and lymph-node imaging [25,29].

Iron oxides vary significantly in color (Table 1) which can help for their identification. They also differ on their oxidation state or oxidation number and their composition, their crystallographic structures, and their magnetic properties. Hematite α-Fe_2_O_3_ is the oldest iron oxide known; it is kinetically and thermodynamically stable. Maghemite γ-Fe_2_O_3_ is on the contrary only kinetically stable, which means metastable, and turns slowly into a stable iron oxide form (hematite) leading to a drastic drop of magnetization. Both vary from red to brown to grey depending on α- or γ- Fe_2_O_3_ (Fe_2_^(3+)^O_3_^(2−)^) polymorph Magnetite Fe_3_O_4_ or FeO. Fe_2_O_3_ is the black iron oxide, commonly called Hercule stone, presenting the strongest magnetic behavior. It is composed of iron(II) and iron(III) and belongs to the category of mixed-oxide (Fe^(+2)^Fe_2_^(3+)^O_4_^(2−)^ or Fe^(2+)^O^(2−)^.Fe_2_^(3+)^O_3_^(2−)^). Mixed-valent magnetite is so far considered in the literature as a charge frustrated iron oxide due to the distribution of both iron(II) and (III) in crystallographic sublattice sites [5,33,35]. 

As shown in Table 1, magnetite is one of the black iron oxides, wüstite is also a jet black color iron oxide but is a quite rare type of iron oxide. In this way, even though color can help to distinguish one iron oxide to another one, it is certainly better to compare magnetic properties. As presented in Table 2, magnetic property is clearly size-dependent, as magnetite and maghemite exhibit both at room temperature, and superparamagnetism below 6 nm and 10 nm respectively. Above those dimensions, they are in a magnetic blocked-state which means that the magnetic ordering at room temperature is the one followed at the particle scale. In this way, magnetite (≥6 nm) and hematite are both ferromagnetic whereas maghemite (≥10 nm) is ferrimagnetic. Contrary to the other two iron oxide types, hematite does not show SPM ordering below tens of nanometers at room temperature but it is able to change of magnetic ordering to antiferromagnetic below the so-called Morin temperature (~260 K). This change of magnetic ordering can be suppressed by a decrease of crystallinity and/or of size NP (≤10–20 nm). Regardless of Morin transition, it is possible to recognize ferromagnetic hematite to ferromagnetic magnetite despite their similar magnetic ordering, as magnetite exhibits the highest magnetic saturation (300 times higher than hematite saturation) [5].

Applications of this group of metallic ferrous nanocrystals can vary quite significantly and are extended, ranging from nanomedicine, as in reviews such as those reported by Laurent et al. [36] or Mahmoudi et al. [28], to catalysis disciplines as shown in Schüle et al. [37], to performing styrene synthesis over iron oxide catalysis or even to water-detoxification as presented by Girginova et al. [38] using a ferrofluid of silica coated magnetite NPs for mercury removal from water. To the best of our knowledge, interest in iron oxide nanocrystals in biomedical fields started a few decades ago and continues growing as a very interesting approach. Consequently, we have made significant progress to figure out the advantages and drawbacks of SPIONs for in vivo uses. However, even though they belong to the group of magnetic nanomaterials, they possess other qualities setting them apart from random magnetic nanostructures. Indeed, in addition to being nanoscale materials which confers the ability to become close to biological entities and enhances interaction with them because of a high surface-to-volume ratio, SPIONs possess the asset of being non-toxic and easily controllable from a distance by an applied magnetic field, and are so far highly suitable for in vivo magnetically-driven treatment [6]. For instance, tumors are treated by hyperthermia process based on the use of magnetic NPs driven by an external magnetic field and then accumulated in injured tissues. Magnetic stimulation can cause an increase in NP temperature up to 42 °C, leading to the death of tumor cells by cellular necrosis, after several minutes of exposure to a radiofrequency magnetic field. Damage of regular tissues, generally occurring as a side effect in cancer treatment, located near injured cells, would not happen by applying such therapy within reason to their natural resistance to heat [2,6]. 

The main requirements when magnetic NPs are employed in biomedical applications, are to use SPM nanostructures exhibiting the highest magnetic saturation and the least toxic components. These two requirements constitute arguments that make scientists think SPIONs are more promising for in vivo applications than other magnetic materials. It should be remembered that superparamagnetism is size-dependent and can be enhanced by an increase of size of NP (without reaching multi-domain nanostructures). Despite a probable improvement of magnetism through using larger SPM NPs, it appears to be contraindicated to employ too large of magnetic nanostructures to ensure a successful diffusion of SPIONs through a limited sized access area in the human body [1,4,22]. As a result, it seems quite clear that working on SPIONs is a dilemma where a balance between the biodistribution and the magnetic properties has to be found for in vivo experiments. In this way, and as for all kinds of nanoscale magnets, researchers have been developing plenty of methods for SPIONs synthesis allowing an accurate control of the size of the produced NPs.

### 3.2. The Need of Surface Functionalization and Stabilization of Iron Oxide Nanoparticles

**Figure 6:**
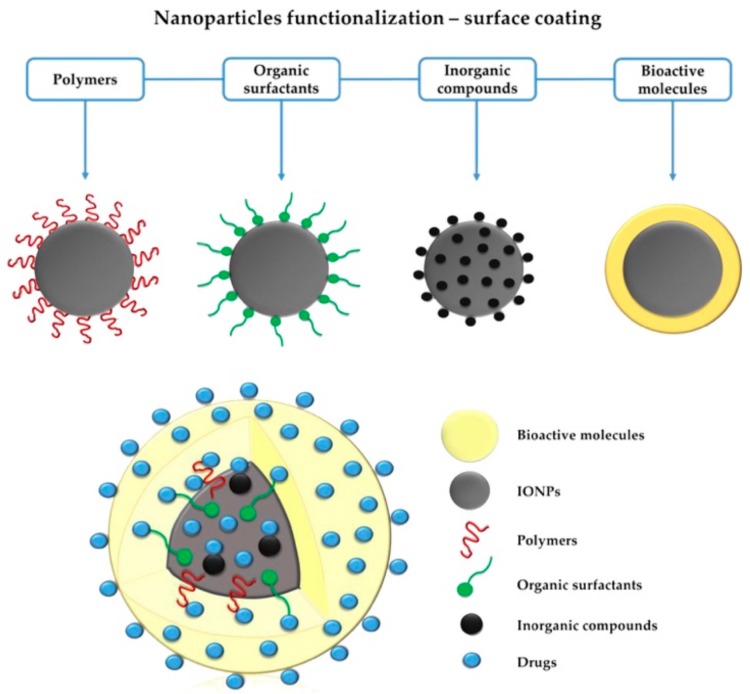
(Top) Schematic representation of the main strategies followed to modify the surface of SPM (iron oxide NPs (IONPs)) (SPIONs). (Bottom) schematic representation of complex structure based on the SPIONs surface functionalization, including drug bounding (Reproduced from Arias, S.L. et al., Antibiotics; published by MDPI, 2018. from ref [39]).

However, even though dimensions of the produced NPs constitute an important parameter to manage, it appears in the literature that iron oxide nanocrystals get a coating corona or ligand layers (Figure 6, [39]) not only to be turned into more biocompatible and stealth nanoscale material but also to be capable of being selectively attached to specific biological entities, to be well-dispersed or solubilized and to be protected from conditions of the media where they are dispersed (oxidation and erosion by acids or bases [1]).

Indeed, magnetite is easily oxidized to maghemite in ambient conditions and requires a corona protection to remain into the magnetite phase. It turns out that several core-shell or -ligand layer constructs have already widely been described in the literature and are mainly based on organic and/or inorganic stabilizing agents. For instance, Liu et al. [40] produced a study indicating that surface modification could lead to better NP stability, enhance NP biodistribution, and increase blood circulation time due to a proper balance between hydrophilic/lipophilic and stealth properties of the outer layer capping the nanomaterial. It has been reported that polyethoxylated amphiphilic compounds are very often used because of their anti-fouling nature and their ability to bypass natural barriers. Gold or silica outer layers represent as well an interesting alternative to pH-sensitive properties, to enhance hydrophilic properties or to provide an easy tailorable surface to graft bioactive materials for targeting [5,11,29,36].

**Figure 7:**
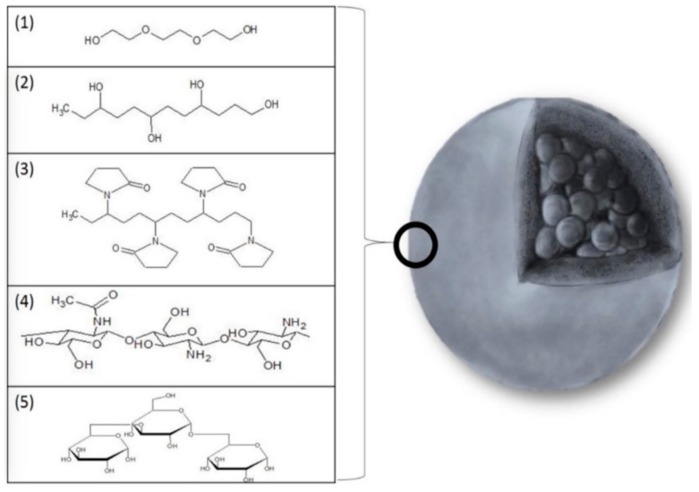
Illustrations of the main compounds used for the surface modification of SPIONs: (1) PEG (poly(ethylene glycol)), (2) PVA (poly(vinyl alcohol), (3) PVP (poly(vinyl pyrrolidone), (4) chitosan, and (5) dextran (Reproduced from Dulińska-Litewka, J. et al., Materials; published by MDPI, 2019. from ref [42]).

Moreover, iron oxide nanocrystals have a high surface-to-volume ratio and thereby a strong tendency to aggregation, leading to the formation of clusters which is quite contraindicated for all biomedical disciplines. Thus, NPs stability needs to be controlled. Derjaguin, Landau, Verwey and Overbeek have long time ago proposed a theory (DLVO theory) about colloid stability and brought the idea that the stability of particles dispersed in solution was relative to electrostatic repulsion and Van Der Waals attraction. However, taking into account the nanosize and magnetic properties, four forces eventually render iron oxide NPs stable or unstable in suspension: as mentioned (i) electrostatic repulsion and (ii) Van Der Waals attraction; and (iii) magnetic dipolar force and (iv) steric repulsion [28,36]. Attractive Van Der Waals forces are mostly the cause of aggregation of NPs because clusters have a lower interfacial energy compared to NPs due to a smaller total area. As has been implied, the surface charge is also involved in the colloidal stability of iron oxides. Even though positively charged NPs hold a good potential for interacting with negatively charged natural entities, this does not correspond to a selective sticking and could occasionally render particles unstable in aqueous physiological environments owing to potential neutralization by the electrolytes existing in blood. A neutral surface is therefore the most promising surface state to minimize nonspecific binding and to avoid a decrease in stability. The zeta potential value (or also Debye-Huckel length related to the screening-charges layer surrounding water-dispersed colloids) is consequently an important parameter to check when NPs are designed to be stable in aphysiological environment and could predict their stability in similar conditions of pH and temperature. Steric repulsions can also play a significant role as a stability factor since the attachment of molecules/ligands onto surfaces can lead to NPs repulsions. In situ or post synthesis polymeric coating (chitosan, polyvinyl alcohol, alginate, dextran, and polyethylene glycol [36,41]) (Figure 7, [42]) provides a steric barrier to the polymer conformation and its chain length, but others criterion can alter its stabilization effects (biodegradation, weak bonding, ability to cover NPs surface…). However, no quantitative values can predict how grafted polymers would dictate SPION stability.

Other routes than in situ functionalization may be applied to adjust specific surface features. A ligand exchange method with biocompatible ligands has been reported as an approach to overcome iron oxide surface failure. Although, most iron oxide syntheses achieved the preparation of hydrophilic materials, a need for a more tunable surface may lead to an exchange capping layer [11]. In this way, to prevent SPIONs from a loss of physical and chemical stability, two types of modification strategies have widely been explored in order to preserve iron oxides from the impacts of the external and in vivo medias [13,28,36,43].

**Figure 8:**
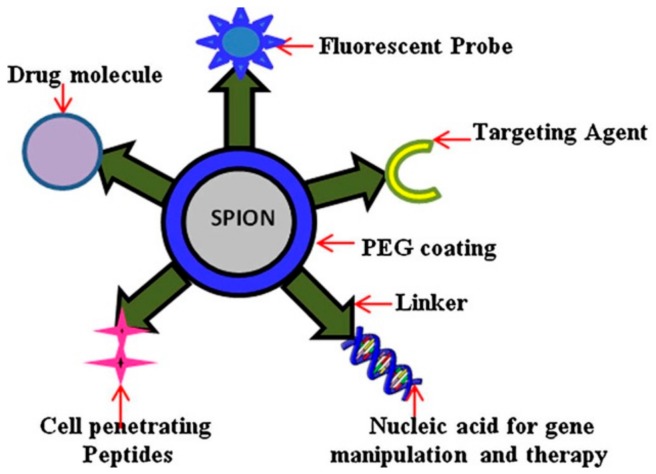
Illustrations of the targeting ligand that can be used to decorate the SPIONs surface: peptides, nucleic acids, and fluorescent dyes (Reproduced with permission from Santhosh, P.B. et al., Cancer Letters; published by Elsevier, Inc., 2013. from ref [44]).

However, it has to be mentioned that a coating strategy provides a core-shell structure and consequently provokes an enlargement of the final magnetic nanostructure. Hopefully, this change is generally about a few nanometers which cannot be considered as a problem for being driven through a limited size access area. What is more, a core-shell structure can confer novel and specific physical and chemical properties to SPIONs thanks to further functionalization which can be done on the corona layer. This approach would provide high effective NPs or nanobiomarkers [25] for selected biological entities. For example, thanks to an approach of surface functionalization (Figure 8, [44]), accumulation of SPIONs within target tissues, like cancer cells, is currently a prominent topic for hyperthermia cancer therapy [22,43]. Such specific and local accumulation in injured cells mostly occurs because a targeting strategy, so called active targeting, has been applied. Injured tissues, generally constituted of abnormal vascular structures, are subject to extravasation, as previously mentioned, but also to a phenomenon of high permeation and retention, referring to the enhanced permeability and retention effect (EPR). This effect renders abnormal tissues more easily able to uptake and accumulate nanoscale entities like NPs and thereby provides passive targeting. Passive targeting cannot prevent accumulation within regular cells, which may lead to cytotoxic side effects. On the contrary, active targeting can avoid a non-selective accumulation and thus avoid some of the damage to regular tissues [13].

### 3.3. Effect of Iron Oxide Design on Magnetism

Nevertheless, applying a surface modification strategy can impact the magnetic behavior, which implies a small enhancement; in contrast, a decrease depends on the outer layer composition [1,29]. When surface modifications refer to the coordination of organic ligands, it has been shown that their adsorption onto magnetic NPs can influence the magnetic saturation. As Daou et al. [45] have proposed in their work about magnetite NPs, the nature of a coupling agent of grafted ligands would not have the same impact on the SPIONs spins. Even more, physorption or chimisorption would also differently impact spin orientation. For example, the chimisorption of a phosphonate coupling agent does not affect the spins at all, whereas the physisorption of carboxylate ligands enables modification of the colinearity of spins along the boundaries of the single magnetic domain. This latter phenomenon is known as the spin canting effect and can make a core-shell material become assimilated to a (core-shell)_a_-shell_b_. Indeed, the existence of a coating corona or adsorbed-ligands (shell_b_) causes a non collinear arrangement of spins along the core’s boundaries (shell_a_), due to interactions between spins from shell_a_ and shell_b_, compared to collinear arrangement within the deep core (core_a_). Consequently, the initial core is no longer magnetically uniform regarding to its boundaries and its deep structure, and that is why it can be associated to an inner core-shell structure ((core-shell)_a_) [46]. Moreover, the crystal morphology of magnetite nanocrystals could also affect its magnetic behavior and provoke an increase of coercivity from spherical and cubic shapes to an octahedral shape indicating an enhancement of magnetism following this series of morphologies. Like the size, magnetic crystal morphology can be controlled with synthesis conditions [5,47].

Synthetic conditions may influence magnetic properties because the structure may be different from one method to another, and also because of the potential presence of a dead magnetic layer like (shell_a_). Regarding the size of the iron oxide particles—which is directly related to the synthetic pathway—magnetic saturation may differ from NPs to bulk materials. For magnetite iron oxide, a decrease of two- or three-fold can be observed for the magnetic saturation from bulk to NPs. Such a difference results in surface curvature of NPs. The smaller the particle is, the more the crystal orientation will be disordered [26]. The effect of disordering in the crystal orientation can be also observed between magnetite and maghemite. Indeed, maghemite is a less symmetric crystal than magnetite which leads to a decrease of magnetism (as shown Table 2).

### 3.4. What About the Consequence After In Vivo Injection of Iron Oxide Nanoparticles?

For real-time biomedical imaging, referring to MRI, the involvement of SPIONs needs some requirements. Indeed, the NP size impacts the contrast greatly, and that impacts the quantity which needs to be injected to patients and the ability of the human body to excrete the substance [29]. It should be mentioned that nanoparticulate contrast agents are basically composed of three main parts: (i) a signal enhancement core, (ii) a biocompatible agent, and (iii) a shell and functional chains grafted onto the core-shell nanostructure for targeting purposes. Most iron oxides NPs are excreted by the liver, due to a selective uptake by the Kupffer cells composing the tissue of this organ. The spleen, the kidneys and the bone marrow are also involved in the metabolism of NPs, as mentioned above with RES. Owing to a fast uptake, a fast excretion from the human body rapidly occurs, which leaves a short period for SPIONs to carry out the purpose they are injected for. For instance, such a rapid metabolic response can consequently induce a lack of accuracy in diagnosis in a case of real-time imaging. Such an excretion process, commonly referred as an opsonization process leading to recognition and clearance mechanism performed by RES, is actually compromising for any kind of NPs but as mentioned above, several factors (size, morphology, surface state…) can be controlled to overcome that fast excretion issue [13].

**Figure 9:**
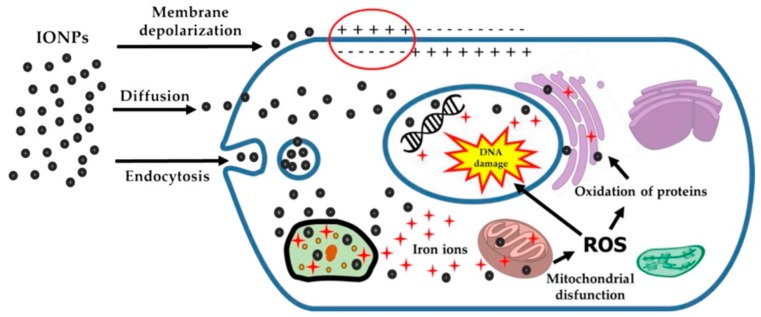
Illustrations of the potential impact of SPIONs on cells, and the toxicity potentially induced (ROS: reactive oxygen species) (Reproduced from Arias, S.L. et al., Antibiotics; published by MDPI, 2018. from ref [39]).

When iron oxide crystalline forms are used for in vivo applications, the human body is able to metabolize them into iron ions and uses the iron released for biological processes such as for red blood cells [11,34]. However, NPs should not be subject to unexpected iron release leading to an increase of iron quantity that would be added to the quantity already existing in the blood stream and causing an overload of iron [32]. Such an unbalanced iron homeostasis could provoke a cellular response. Although employing SPIONs seems so far to be quite safe, due to the significant progress made to adjust them or to make them fit for in vivo applications, an exposure to a high dose can induce potential cytotoxicity, impair the functions of some major entities involved in human biological systems, including DNA and mitochondria, and cause mild and short duration side effects (nausea, urticarial, diarrhea) as globally illustrated in Figure 9, [39].

Considerable efforts have been devoted to designing SPIONs possessing physico-chemical characteristics to produce very low toxicity nanostructures. However, it is still important to carry out a follow-up of human body response in order to prevent the toxicity related to break-down products and the potential interactions between metabolites and tissues where SPIONs are accumulated [34]. In other words, a complete stability study in physiological conditions of iron oxide NPs should be performed for any new designs. This also concerns consequently functionalized core-shell structures, as the involvement of biocompatible polymers, organic, or inorganic shells like silica require a toxicity study in response to current debates about those compounds and their effects on health [11].

Consequently, it is challenging to produce desired SPIONs, exhibiting optimal morphological dimensions, good magnetism behavior, high stability and dispersibility, to form a selective biocompatible colloidal suspension in a physiological environment. Those characteristics are certainly parameters that have to be fully controlled and some others might have to be deeply investigated, like the toxicity. However, magnetism remains also an important parameter. Indeed, to as SPION magnetism can easily be altered it should briefly be noted how it can be affected: It is quite dependent on (i) the composition and consequently (ii) the crystallographic structure (magnetite, maghemite and hematite), (iii) the particle size (single domain or multi-domains), and (iv) the surface state (coating or ligands).

Among all iron oxide nanomaterials, maghemite and magnetite are very popular and promising nanocrystals in biomedical disciplines [43]. Additionally, Table 2, inventorying magnetic saturation, indicates that magnetite is the most magnetic type of iron oxide. It appears that it corresponds also to the iron oxidethat is most sensitive to oxidation [5] and inevitably needs a protective capping layer. Yet, magnetite seems increasingly attractive due to its large magnetism and the huge development of functionalization after an adapted chemistry of surface.

## 4. Preparation of Magnetite

### 4.1. Formation of Iron Oxide Nanoparticles

**Figure 10:**
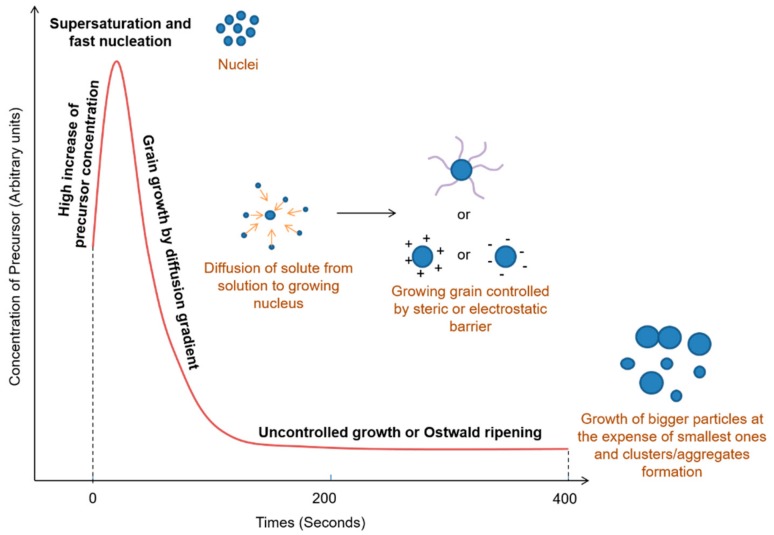
Illustration of the steps of formation of NPs initiated by an increase of precursor concentration.

As has been widely aforementioned, the design is a key parameter for in vivo applications and governs the in vivo fate of SPIONs. Biomedical disciplines require high quality magnetic NPs, i.e., narrow size distribution, stability in physiological environments (weak aggregation tendency, no premature degradation and release of material, stealth surface properties), biocompatible components, specific accumulation by vectorization or simple passive targeting, etc. and that is regardless of magnetic properties. Most of these features can be adjusted during NPs formation thanks to appropriate conditions of synthesis. Typically, SPIONs like magnetite are produced via a two-steps mechanism by homogeneous precipitation, i.e., a single liquid phase process. This two-step mechanism was proposed by LaMer et al. [48] and was completed by other authors like Ng et al. [49]. However, as yielding monodisperse NPs is actually related to several parameters, the main conditions to control are the concentration of precursor involved into the reaction, the amount and the nature of ligands, and the temperature. Gathering optimum conditions lead to the formation of SPIONs as described in Figure 10. Essentially, it begins with the supersaturation of the solution in solute, this phenomenon can be done by a high increase of solute concentration thanks to their solubilization in the reaction media and/or an increase of temperature. This leads to a fast second step of nucleation corresponding to a burst nucleation and providing tiny crystalline nuclei. Nucleation is an endothermic process due to the splitting bonding of reactants, desorption of the solvent shell from compounds and so counteracting surface tension consuming energy. Afterward, an exothermic step follows and is assimilated to nuclei growth (third step) where those grains grow by the diffusion of solute from the solution towards the grain surface. This step is actually energetically favorable since it reduces the surface-to-volume ratio of particles. The growth step can be kept under control since the surfaces of iron oxide grains are generally covered by surfactant, organic ligands, or other electrostatic or steric stabilizers, preventing polydispersity issues from happening. To sum-up, monodisperse NPs can then be obtained by a high initial supersaturation level forcing the system to incorporate solute to growing nuclei. In addition, clusters, aggregates, or bigger NPs might be formed in the case of uncontrolled reaction conditions, in some cases, Ostwald ripening may occur and can then be assimilated to another step of nucleation (fourth step) [4,12,28,36,41,50].

As mentioned, the temperature is also a key factor to control the formation of monodisperse SPIONs because it can help to differentiate nucleation to growth steps. Zhao et al. [51], suggested a thermal decomposition of an organometallic complex of iron in methoxy polyethylene glycol (MPEG), and assumed that nucleation is a process occurring around 200 °C whereas growth happens at higher temperature around 240 °C. They also reported that the formation of nuclei would be initiated by the reaction of ferric ion and a hydroxyl group from MPEG leading to partially oxidized MPEG-coated iron oxide magnetite nuclei.

This mechanism of formation has been introduced for a single phase procedure, however, it has been reported that SPIONs can be prepared by a two phase procedure, like the aerosols methods or laser pyrolysis, involving gas and liquid phases. Nevertheless, the as-produced iron oxide nanocrystals are mostly based on maghemite and hematite iron oxides which correspond to less magnetic iron oxide types. In addition, such procedures induce a decrease of magnetic saturation compared to a homogeneous precipitation technique. Moreover, scaling-up remains challenging and the conditions to optimize the reaction yield and the purity are not fully managed yet [5,50]. Consequently, we will now focus on a pathway to synthetize the most magnetic iron oxide, the magnetite Fe_3_O_4_. In order to figure out which technique yield suitable design of SPIONs depends on the desired applications, an overview of synthetic methods will be presented hereinafter.

### 4.2. Synthesis of Magnetite SPIONs

The challenge of producing SPIONs for biomedical uses is to find a method allowing a high amount of very low toxic and monodisperse NPs by, if possible, an ecofriendly and scaled-up route. In addition, some specifications related to the design may be necessary to fulfill depending on the biomedical applications.

Syntheses of magnetite SPIONs using microorganism routes with bacteria (magnetostatic bacteria) or fungi have previously been discovered [28,43]. In 2005, Bharde et al. used *Actinobacter spp.* bacteria [52] and *Fusarium oxysporum* and *Verticillium sp.* fungus [53] for that purpose. Currently, it is more common to work with chemical methods via an iron precursor reduction (metal salt or organometallic complex) due to the need for well-designed NPs for large-scale synthesis [28,43]. Researchers have focused their efforts on two main pathways to produce tailorable and monodisperse magnetite SPIONs. Among all techniques developed, we can mention the coprecipitation, the high temperature decomposition, the constrained environment procedures, the polyol method, the hydrothermal method, the sonochemical procedure, and the electrochemical methods; coprecipitation and the high temperature decomposition, commonly called thermal decomposition or pyrolysis, have been widely applied to synthesize magnetite during the last decade. Many reviews [1,5,26,28,36,41,43,50] have reported the particularities of each chemical technique and have indicated that coprecipitation and thermal decomposition correspond to the scale-up pathways in reason to the reproducibility and the production of monodisperse nano-sized magnetite particles. Before going deeply into details for those two kinds of synthesis, a brief overview regarding the other ways remains interesting in order to sum-up some advantages and disadvantages of these methods.

Constrained environment procedure: To begin with constrained environment reactions, we have to distinguish three main systems: microemulsions, dendrimers, and liposomes constructs. For procedures involving dendrimers or liposomes, the organic structures of dendrimers and liposomes are used as template hosts and provide generally magnetodendrimers and magnetoliposomes owing to the encapsulation of SPIONs within their cores [41,50]. The microemulsion method has been more detailed in the literature. Microemulsion structure involves iron oxide synthesis mostly as a reverse emulsion corresponding to a water-in-oil (W/O) suspension. Such a colloidal suspension is based on a stable dispersion of two immiscible liquids in a shape of microdroplets (1–50 nm) of an aqueous phase, containing reactants, surrounded by an interfacial film of surfactant molecule assemblies and dispersed in an oily phase. The confinement of reactants within droplets is the key of monodispersity since it prevents NPs produced in situ from aggregating and also allows accurate control over the size range by adjusting conditions like the temperature of formulation, the surfactant concentration, and the flexibility of the interface. Microemulsion’s microcavities are basically used as nanoreactors since two identical W/O microemulsions, containing respectively one reactant, can be mixed in order to make droplets coalesce to finally provide SPIONs within micelles. The final product can then be extracted from the droplet’s core by breaking down the emulsion, for instance, with hydrotrope molecules (ethanol, acetone…) and then precipitating from the continuous oily phase. Magnetite NPs can be obtained through this process, but the yield remains quite low and a high amount of solvent is required [1,5,26,50]. NPs are also poorly crystallized and smaller than 50 nm. In addition, surfactant molecules can be still present as impurities from the reaction onto NPs surface which might contraindicate in vivo use [1,28]. Recently, Lee et al. [54] proposed a compromise to overcome the lack of crystallinity and monodispersity of magnetite NPs prepared within nanoreactors based on reverse micelles. Gathering stable nanoreactor formulation under high temperature conditions with a small amount of solvent, they settled on an ecofriendly scale-up process providing highly crystallized and monodisperse 10 nm magnetite NPs. Their synthesis of SPM NPs was considered an improvement of the constrained environment technique since it involved a small amount of solvent, a high temperature causing enhancement of crystallinity, and corresponds to a one-step procedure in reason to production of monodisperse NPs without post-synthesis size-selection.

Polyol method: Polyol method is also a convenient method to prepare magnetite NPs due to the possibility of obtaining NPs with a narrow size distribution. In this kind of procedure, polyol plays many roles including the solvent of inorganic components, the reducing agent of the iron precursor, the stabilizer during the nuclei growth step preventing the form aggregation, and the protective capping as the hydrophilic ligand adsorbed onto a magnetite NP’s surface. Essentially, microparticles and NPs are easily dispersed in an aqueous polar media and possess a high crystallinity because of the high temperature, near the boiling point of the polyol, applied during the process. This technique is a one-step procedure since suspension of the iron precursor in polyol leads by an increase of temperature to a solubilization of the precursor and its reduction provides iron oxide nuclei [36,50]. After the growth step, SPIONs precipitate from the solution. Metallic alloys are mostly produced by the polyol method [55,56,57] rather than from oxide alloys but the literature still reported magnetite NP preparation. For instance, Wan et al. [58] reported the preparation of water-soluble magnetite SPIONs for MRI. This work was focused on the finding of a compromise between the ease of the coprecipitation process and the high quality of magnetite NPs provided by thermal decomposition. The non-aggregated and polyol functionalized magnetite NPs were synthetized by reacting iron precursor and iron(III) acetylacetonate, in triethylene glycol near the boiling point of the polyol. The true diameter was found by transition electron microscopy (TEM) close to 10 nm but the hydrated polyol chains provided, by dynamic light scattering (DLS), a hydrodynamic size near 16.5 nm. The magnetic saturation was 80 emu·g^−1^ suggesting that NPs magnetically behaved mostly like a bulk material using a high temperature process. It seemed quite clear that working a under high temperature may help to get high crystalline nanomaterials leading to high magnetic magnetite nanomaterials. Combining nanoscale dimension and high magnetism, the magnetite NPs were suggested to have proton relaxation time measures, revealing their potential as a contrast agent for MRI (r2 = 82.86 mM^−1^·s^−1^).

Hydrothermal technique: The hydrothermal process is referred to as the oldest method used for preparing uniform nanostructures like magnetite NPs and composites. It is based on the ability of water to hydrolyze and dehydrate metal salts under harsh conditions of pressure and temperature. Those two parameters are the keys to control nucleation and growth of iron oxide nuclei. Owing to the absence of an organic solvent, the hydrothermal procedure is eco-friendly, however the main disadvantage is the impossibility to functionalize in situ NP surfaces. However, it appears to be a funtional method to obtain highly crystalline NPs with well-defined morphology and size. Finally, if the process is fully managed, the magnetic property will be optimized, indeed, magnetic saturation of NPs would be quite close to that from bulk materials which is a very rare property [1,5,28,59]. Daou et al. [60] worked on magnetite synthesis and showed that post-synthesis hydrothermal treatment allowed the recovery of magnetite with an unoxidized surface. They proved in this work that slight deviation of the stoichiometry on magnetite grain surfaces, corresponded to the maghemite layer, and lead to a decrease of crystallinity and so a decrease of magnetic properties. However, studies have been done on this point where they showed that the time of the reaction can overcome this issue and decrease the ratio of magnetite to maghemite by increasing the grain size [61]. With hydrothermal treatment, almost whole nanocrystals were constituted of pure magnetite and exhibited a higher magnetic saturation than before treatment. It seems that the hydrothermal method might be more interesting to perform as post-synthesis treatment if NPs display a high degree of oxidation. Recently, novel hydrothermal techniques have emerged and combined coprecipitation synthesis and thermal treatment to provide theranostic SPIONs dedicated to anemia treatment and MRI biomedical applications. Such a combination yielded nanodrugs with enhanced crystalline structure, e.g., enhanced magnetic properties [62].

Sonochemical procedure: It involves a high energy ultrasonication to create acoustic cavitations into a liquid media causing the formation and the growth of bubbles containing the reactant in a gas state. This leads to a local increase of pressure close to 1800 atm and hot spots at around 5000 K, making chemicals in the gas state react and bubbles collapse. The sonochemistry applied to SPIONs, allows materials with unusual properties like a low magnetic saturation for magnetite and different shapes. As mentioned, the sonochemical method induces a very high increase of pressure and temperature rendering this process difficult to be managed and inappropriate for scaled-up production [28,43,50]. Vijayakumar et al. [63] reported a sonochemical procedure to prepare 10 nm Fe_3_O_4_ SPIONs with a very poor magnetism, like amorphous particles do, despite the crystalline structure. So far, sonochemical synthesis provides generally amorphous materials, or a non-crystalline phase, due to the quick transition from harsh conditions to standard ones.

Electrochemical method: This is certainly the less applied method to prepare SPIONs for biomedical applications. Few details are available in the literature, however it has been identified as a method providing magnetite and maghemite SPIONs. The electrochemical pathway involves an anode and a cathode, the first electrode, a sacrificial electrode, gets oxidized into a water-soluble metallic species which is then reduced onto the cathode in metal in presence of stabilizers. Even though it is not a widely applied technique, it allows obtainment of NPs with a good control over the size by a direct control over the current density [28,50]. Cabrera et al. [64] achieved by iron electrooxidation the synthesis of 20–30 nm spherical ferromagnetic Fe_3_O_4_ NPs and suggested that synthesis was dependent to the distances between electrodes which must be short enough to make sure that species will be able to reach both electrodes, the pH to optimize reactions involved, and the potential to avoid production of gases (H_2_ and O_2_) on the electrode surface, preventing complete reaction from occurring. In addition, the authors exposed the impact of current density on polydispersity. As aforementioned, redox media led here to maghemite layer capping of magnetite NPs, indicating oxidation issues during the process.

Coprecipitation and thermal decomposition possess many advantages. Nevertheless, different disadvantages can be pointed out in the literature. They have at least a common point of being considered as scale-up techniques. Many arguments have been debated about those two procedures regarding the size distribution, the conditions of preparation (solvent, temperature) and the surface property of the SPIONs. For example, it is assumed that coprecipitation would provide nanomaterials with a quite large size distribution leading to a non-ideal magnetic behavior compared to the uniform size NPs obtained from thermal decomposition. Indeed, within a batch of nanomaterials produced by coprecipitation, several monodisperse populations of particles would coexist and would be magnetically inequivalent regarding the blocking temperature. So, instead of exhibiting a unique temperature of transition, as an entire batch produced with a very narrow range of size, a polydisperse batch would have a range of blocking temperatures since each population with a given size of NPs would have its own blocking temperature. However, post-reaction size selection can be performed to reduce the range of sizes [1,43]. 

The positive side for coprecipitation relative to thermal decomposition is that coprecipitation is a simple, ecofriendly, and solvent-free process giving water-soluble and biocompatible SPIONs [5,41]. For imaging applications like MRI, it is imperative to use stable colloidal suspensions and non-toxic NPs in physiological conditions [11], so far this seems achievable by coprecipitation. The dilemma here is about the quality of the colloids from the coprecipitation reaction as it has been pointed out. High quality SPIONs are mostly produced by thermal decomposition. High quality NPs refer to highly crystalline NPs based on a single monodisperse population with a well-defined magnetic property. However, a thermal decomposition procedure involves organic solvents and lipophilic stabilizers whose use might be detrimental for the biocompatibility. In addition, SPIONs from high-temperature decomposition are consequently mainly hydrophobic and so insoluble in aqueous or polar solvents [1,5,11,41,43,50].

Coprecipitation: Coprecipitation corresponds to precipitation of substances that are normally soluble and which can be carried out by two pathways to obtain magnetite NPs in alkaline aqueous media: (i) the partial oxidation of Fe^(2+)^ salts or (ii) the aging of a stoichiometric mixture of Fe^(3+)^ and Fe^(2+)^ salts [50]. Though, all procedures assimilated to the conditions of (i) turn into (ii) since Fe^(2+)^ gets slightly oxidized, generates Fe^(3+),^ and forms a mixture of both iron salts. The general reaction principle is essentially the following one [65]:

(3) $${\mathrm{Fe}}^{\left(2+\right)}+2{\mathrm{Fe}}^{\left(3+\right)}+8{\mathrm{OH}}^{\left(–\right)}\Leftrightarrow \mathrm{Fe}{\left(\mathrm{OH}\right)}_{2}+2\mathrm{Fe}{\left(\mathrm{OH}\right)}_{3}\to {\mathrm{Fe}}_{3}O_{4}+4H_{2}O.$$

Several parameters have to be carefully mastered to keep a control over the range of sizes, the colloidal stability, and the oxidation surface of the produced nanocrystals of magnetite. Those have been widely studied due to the increasing interest of researchers on the simple procedure of coprecipitation. First, the reaction might be done in an oxygen-free environment to prevent the formation of magnetite NPs from oxidation, and second it should involve hydrophilic ligands in situ (dextran, polysaccharide, polyvinyl alcohol, starch, hydrophilic polymers…) to stabilize and avoid any unexpected aggregation by a quick adsorption right onto the just formed nuclei [1,41,50]. Surface composition and charge are ispo facto related to the type of ligands adsorbed onto NPs, however those are important for control over the growth step of nuclei. Third, regarding charges, it should be mentioned that coprecipitation is basically performed in water suggesting the presence of electrolytes. The pH, the ionic force, and counterion of the metallic salts (and consequently the nature of the metallic precursor) are involved to stabilize NPs. Indeed, all of these parameters play a role in the surface state of the NPs in an aqueous suspension since controlling them can lead to the formation of a less compressed electrostatic double layer (Stern layer) and so generate an efficient electrostatic barrier preventing coagulation [5]. Consequently it has been identified as important to work in the case of (ii) with a Fe^(2+)^/Fe^(3+)^ ratio of 0.5 at a pH around 8–14 for performing a reaction in favorable thermodynamic conditions to obtain magnetite [41]. Finally, the temperatures of the reaction are also important for the size. Like in the polyol procedure and thermal procedure, it appears to work better close to the boiling point of an aqueous solvent or a polar solvent [1].

A typical example highlighting the importance of the reaction conditions to control the design of SPIONs is the study performed by Aslam and co-workers [66]. They worked on the synthesis of uniform water-dispersible magnetite nanoparticles surrounded by amine ligands from a single precursor, FeCl_2_. They indeed showed the influences of molar ratio between dodecylamine and iron precursors and reaction time on the size distribution and the magnetic properties like magnetic saturation and transversal relaxivity (r2). Their results suggested that working in a high amount of amine (from 1:1 to 7:1) would provide smaller magnetite nanocrystals (from 40 to 8.5 nm) with a narrow size distribution by reacting over a long reaction time (from 3 to 12 h) and with good properties as a T2-weighted MRI contrast agent (r2 from 80 to 232 mM^−1^·s^−1^). Another case showed also that coprecipitation can easily be a green process and yield biocompatible SPIONs. Lu et al. [67] reported a simple procedure involving FeCl_3_ and α-d-glucose for its reducing property providing hydrophilic magnetite SPIONs functionalized by the oxidized form of glucose, the gluconic acid. By applying a simple green chemistry synthesis at 80 °C, they generated 12.5 nm uniform and spherical magnetite nanocrystals exhibiting a magnetic saturation close to 60 emu·g^−1^. The same team introduced a parallel work using the as-introduced magnetite nanoparticles as a seed template for selenium corona growth to extend its applications from drug carrier [67] to further functions in the biology and medicine fields [68].

To go further into the understanding of impacts of reaction parameters over SPIONs design and their resulting properties, we can mentioned Lyon et al. [69] using coprecipitation inspired from Kang et al. [70] and a gold coating by an iterative procedure to yield a magnetite core with a maghemite layer and gold corona shell. Here, the oxidized surface has been pointed out as an asset to easily coat the grain surface with gold due to a better reduction of Au^(3+)^ to Au^(0)^ onto the maghemite surface. It was reported that magnetism would have not have been affected by gold deposition; however magnetic saturation remained quite low for a magnetite core (45 emu·g^−1^). Although no applications were cited for future uses of the assembly, this construct could hold potential for X-ray/MRI bimodal imaging since it appeared like a well-designed water-soluble and nanoscale material for this end. Gold coating is certainly the most applied gold capping, whatever the synthetic method used to make magnetite nanograins. Some authors then worked on the attachment of gold clusters to 10 nm SPM biocompatible magnetite to overcome the potential alteration of magnetic behavior due to the thickness of gold coatings. Caruntu et al. [71] synthesized magnetite by coprecipitation and attached 2–3 nm gold clusters onto the as-prepared SPIONs. In this case, the Fe_3_O_4_–Au nanocomposites exhibited a magnetic saturation near bulk material, indicating the absence of impact of cluster attachment in contrast to gold coating in the previous study. 

Sun et al. [72] also introduced the impact of biocompatible ligands on magnetite nanoparticle behavior. Surfactants, sodium oleate and polyethylene glycol, were respectively implied as surface grafted ligands. Experiments allowed them to conclude that the sodium oleate was the best candidate to optimize the magnetic saturation of the coprecipitated magnetite, the biocompatibility, and the dispersion in aqueous media. To go deeper into the feasibility of tuning the magnetite from coprecipitation, Wang et al. [73] achieved the first fabrication of a multifunctional assembly based on the combination of the layer-by-layer self-assembly method and dendrimer chemistry on magnetite iron oxides nanoparticles from coprecipitation to constitute a platform for MR imaging and cancer cell targeting. Positively charged magnetite NPs were subjected to the addition of negatively charged polyelectrolytes and then to dendrimer formation by the layer-by-layer technique. The outer layer of the dendrimer was prefunctionalized with moieties, based on folic acid, specific to receptors over-expressed onto cancer cells for targeting ends and to avoid any unexpected sticking to random in vivo biomolecules. The magnetic core showed good potential for T2-weighted contrast for MRI. Wang et al. [73] introduced a novel route for cancer cell imaging by applying a biocompatible targeting strategy with dendrimer surface design encapsulating SPIONs with enhanced contrasting properties.

As can be noticed, coprecipitation is widely applied owing to its simplicity and capacity to yield SPIONs with satisfying properties for in vivo uses by involving biocompatible and water-soluble components. In addition, this brief overview of examples can also be used as a proof of concept concerning the importance of the surface chemistry of magnetite not only for the desired application but also to tune magnetic properties.

Thermal decomposition: The three iron oxide nanoparticles can be produced by this high temperature process under different temperatures of reaction: the higher the temperature is, the more oxidized the iron oxide will be. Basically, the thermal decomposition lays on the decomposition of an iron organometallic precursor by high temperature via the use of a high boiling point organic solvent (phenyl ether, or diphenyl ether) in the presence of a surfactant (fatty acids, fatty amine) introduced in a specific ratio compared to the iron complex. Many organometallic complexes are used, the most common are divided up in three families: (i) iron carboxylate (iron acetylacetonate Fe(acac)_3_, iron oleate Fe(oleate)_3_), (ii) iron carbonyl (iron pentacarbonyl Fe(CO)_5_), and (iii) iron cupferronate (Fe(Cup)_3_) [1,4,5,41,43,50]. Deep investigations about those three families have been done over the two last decades and have led to identify (ii) and (iii) as iron precursor families mostly applied for preparations of γ-Fe_2_O_3_ maghemite nanoparticles. Regarding the most referenced works, it appears clear that Fe(CO)_5_ and Fe(Cup)_3_ were both involved in γ-Fe_2_O_3_ syntheses by thermal decomposition as it was achieved fifteen years ago by Hyeon et al. [74] and Rockenberger et al. [75]. Counter to (ii) and (iii), iron complex (i) have mainly been referred in papers for magnetite nanocrystal synthesis. Regarding major works related to magnetite synthesis by thermal decomposition, Fe(acac)_3_ was used by Sun et al. [76,77] who have established a work on which dozens of authors have then been inspired. In order to pursue this goal, Sun et al. [76,77] reported a thermal decomposition procedure providing hydrophobic magnetite SPIONs from 4 nm to 20 nm (by seed-mediated growth for sizes up to 8 nm) followed by ligand exchange post synthesis procedure with tetramethylammonium 11-aminoundecanoate to turn those lipophilic magnetite nanoparticles into hydrophilic nanomaterials [76]. Nucleation and growth steps were performed by applying two temperatures yielding monodisperse magnetite; oleylamine and oleic acid were used as ligands. In thermal decomposition, surfactants work as stabilizer molecules by acting as steric barrier between particles and inhibit uncontrolled nuclei growth during the process, which is why this method provides dispersed nanoparticles with very well-defined morphology without aggregation issues [1,41].

Numerous researchers have been interested in Sun et al.’s thermal decomposition [76,77]. Many of them inspired their work from these protocols. For examples, Wang et al. [78] produced a core-shell assembly called Fe_3_O_4_@Au based on hydrophobic NPs coated with gold layer by in situ reduction of Au^(3+)^ into Au^(0)^. Gallo et al. [79] achieved a recent work on water-soluble gold-coated magnetite functionalized with neoglycoconjugates and a linker for specific biological entity recognition. Magnetic properties was unchanged after the attachment of ligands, thus the ligand-grafted core-shell was expected to be efficient for MR imaging (r2 = 155–157 mM^−1^·s^−1^). Similarly, Garcia et al. [80] made 6 nm magnetite–gold core-shell nanomaterials based on antibody-magnetic glyconanoparticles for immunolabeling of cells. MRI contrast was proven and they showed the potential of the magnetite core of T2-weighted contrasting for cell imaging (r2 = 156.7 mM^−1^·s^−1^). The gold shell was included into this novel structure due to the gold-thiol chemistry offering a range of possibility for further functionalization by reacting the thiol-end of the chain, with antibodies. By applying the same synthetic method, Pandey et al. [81] have recently reported the use of magnetite nanoparticles as biosensors for *Escherichia coli* bacteria.

The use of magnetite produced by thermal decomposition described by Sun et al. [76,77] has also been investigated in the field of multimodal imaging. Preparation of a multimodal probe usable in MR, X-rays, and optical imaging were reported by Dong et al. [82] for cancer diagnosis. Magnetite was chosen to enhance MRI contrast, rhodamine compound was used as a dye for fluorescent imaging and a gold corona layer was involved in a double role as (i) X-ray contrast agent and (ii) a shell easily modified to have an anti-fooling property by grafting thiolated polyethylene glycol polymer by thiol-gold chemistry. Biocompatible nanospheres with 101 nm hydrodynamic diameter were formed with a well-defined shape and a good colloidal stability in physiological media. Focusing on the magnetite core, MR imaging of mice showed significant darkening of hepatic lesions over half an hour indicating a promising ability for contrasting in T2-weighted imaging (r2 = 242 mM^−1^·s^−1^). Carril et al. [83] proposed another version of multifunctional nanocomposites for multimodal imaging based on magnetite glyconanoparticles as a 4 nm SPM core produced with the procedures of Gallo et al. [79] and Garcia et al. [80], for T2-weighted MRI (r2 = 160 mM^−1^·s^−1^) with a gold coating for X-ray contrast. Ultrasound contrast was also investigated for this assembly.

Different types of stabilizers other than oleylamine and oleic acid have also been attached; a new design of magnetite has, in this way, been introduced. For instance, Li and co-workers [84] proposed a thermal decomposition to provide hydrosoluble magnetite nanoparticles by reacting Fe(acac)_3_ and 2-pyrrolidone used as solvent, reducing agent, stabilizer, and coordination agent via carbonyl-iron interaction. It should be notified that this method constitutes an enhancement because water-soluble iron oxide nanoparticles were made in a one-step procedure avoiding as a result the need of applying a ligand exchange procedure. SPIONs (5 nm) were prepared by a one-step reaction, whereas bigger SPIONs (11 nm) were produced by seed-mediated growth. They respectively showed low magnetic saturation of 31 emu·g^−1^ and 65 emu·g^−1^ supporting the impact of an increase of size and a change of surface state on magnetism. Indeed, the difference of ligands covering the nanoparticle surface generated a higher spin-canting effect due to the high polarity of 2-pyrrolidone and consequently a decrease of magnetic saturation.

As to the role of temperature, working in polyol process with a high boiling point solvent is needed to obtain highly crystallized nanoparticles. Larger nanoparticles are also generally produced by working at high temperatures [1,41]. Such characteristics, and also the impact of the ratio between surfactants and organometallic complex, were widely noted in cases of Fe(oleate)_3_ uses. Fe(oleate)_3_ or iron carboxylate salts are mainly involved in procedures including the formation of stable Fe(oleate)_3_ starting materials and its in situ thermal decomposition in the presence of solvents with a boiling point up to 300 °C (octadecene, trioctylamine…). Yu et al. [85] explored that pathway to achieve magnetite SPIONs with a high quality as spherical magnetic colloids. By applying different ranges of molar ratios of oleic acid and an iron precursor to generate Fe(oleate)_3_, different times of reaction, and reaction temperatures, they investigated impacts on nanoparticles size distributions which became broader and broader by increasing the two last parameters due to Ostwald ripening. An excess of oleic acid had caused an inhibition of magnetite nuclei formation indicating the importance of the ratio between reactants and stabilizers. Park et al. [86] attempted a two-step procedure consisting in first place to the synthesis of Fe(oleate)_3_ salt and in second place to the large scale production of monodisperse magnetite SPIONs without size-selection requirement. Authors highlighted the need of distinction between nucleation and growth kinetics by introducing a hypothetic mechanism of formation pointing out the role of the Fe(oleate)_3_ and the temperature. As to the impact of temperature factor, they worked with various solvents to form high crystalline nanoparticles with higher size (9 to 22 nm) due to the higher boiling point (274 to 365 °C). Although, those authors have not proposed applications for the future, Lee et al. [87] have recently been working on thermal decomposition of Fe(oleate)_3_ from Park et al. [86] for multimodal imaging including MRI of tumor allowing to discriminated vascular region of a rat tumor. To conclude, the ratio between the surfactants and the organometallic complex has to be managed as much as the time of reaction and the temperature. Other parameters like the stirring, or even the ratio between the different types of stabilizers involved [88], must be taken account to keep a control over the size, the crystallinity, and the shape to determine enhancement of magnetic properties.

**Figure 11:**
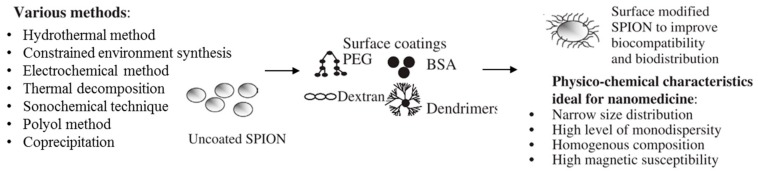
Methods and in situ and/or post-synthesis treatment to form biocompatible SPIONs for nanomedicine purposes (Reproduced from Singh, N. et al., Nano Reviews; published by Taylor & Francis, 2010. from ref [34]. BSA means Bovine Serum Albumin).

Many techniques are described in the literature to yield SPIONs exhibiting suitable features for in vivo uses. Figure 11 summarizes the different methods to obtain SPIONs, and consequently magnetite, how to turn them into biocompatible nano-assembling, and the stringent requirements to consider SPIONs as ideal NPs for nanomedicine.

Although it is important to have an overview of methods to produce SPIONs with suitable properties, it is also interesting to be aware of techniques to determine in an efficient way how to characterize SPIONs to identify, quantify, and define their composition, their design (shape, size, and surface features), their physico-chemical, and magnetic properties. To this end, we can quickly establish a listing of the most applied methods and characterization techniques commonly presented in the literature, which can be divided in several groups depending on the information that they provide: there are i) methods of identification such as spectroscopic techniques by means of infrared spectroscopy (FTIR) [47,50,89,90,91,92], X-ray photoelectron spectroscopy (XPS) [93,94], Mössbauer spectroscopy [3,35,95,96], and X-ray diffraction [5,76,91]; and ii) methods of quantification like relaxometry titration [97], thermogravimetric analysis (TGA) [66,76,98,99], inductively coupled plasma (ICP) [100], and rarely high resolution magic angle spinning (HR-MAS) [101] (XPS can also be included in such kind of characterization due to its principle); there are techniques providing iii) physico-chemical information like dynamic light scattering (DLS) and electronic microscope pictures by transmission electron microscopy (TEM) (morphology, size) [28,40,41]; and finally, iv) magnetic properties (hysteresis loops, magnetic saturation, and coercivity) by superconducting quantum interference device (SQUID) (and also Mössbauer spectroscopy) [102,103] and relaxometry [104]. A complete review could be dedicated to the description of all of these techniques in order to provide a complete guide for SPION characterizations from basic techniques (infrared spectroscopy, X-rays diffraction, DLS, etc.) to the most specific methods (relaxometry, SQUID, and HR-MAS).

## 5. Further Outlook

In addition to the traditional synthesis methods, besides the classical uses of SPIONs as T2-weighted imaging agents, most research efforts are now dedicated to the modification of the magnetic nanoparticles for specific applications. The main important and prevailing purpose in today’s research on SPIONs regards the development of targeted theragnostic solutions. This is due to a real need to prematurely access pathologic regions and provide an early diagnosis of the, e.g., small growing tumors. In the case of tumors, even if compared to healthy tissues, the tumor microenvironments present a number of aberrant characteristics that are often exploited to develop anticancer therapies [105], many strategies have been worked out to actively target the cancer cells. The use of ligands to decorate SPION surfaces is generally considered, such as transferrin [106] or other specific peptides (TWEAK) [107], to target glioblastoma. However, in general, a variety of tumor cell-specific ligands used to decorate the magnetic nanoparticles are reported in recent literature reports, targeting breast cancer cells (with anti-human epidermal growth factor receptor-2 single-chain antibody fragments) [108], squamous cell carcinoma [109,110], and hepatocellular carcinoma (using single-chain antibodies for the epidermal growth factor receptor) [111]. In this latter report, the tumor proliferation was elegantly inhibited through the targeting of nanomedicine that silenced the PBOV1 (prostate and breast cancer overexpressed gene 1) gene (recognized as promoting hepatocellular carcinoma proliferation).

In addition, the theragnostic approach also includes using the SPIONs properties to destroy proliferating cells, by hyperthermia-mediated drug delivery [112], or in combination with optical probes able induce photothermal effects when combined with heptamethine cyanine [109], treating tumors in mice by photodynamic therapy using Chlorine e6 or Dextran Benzoporphyrin coating SPIONs, reported in [113] and [114], respectively.

Multimodal imaging is also a very active research axis still working with SPIONs and developing innovative diagnosis and theragnostic tools. The most common approach is the combination of MRI with optical or near-infrared optical imaging, as these two modalities are very complementary. In fact, near-infrared fluorescent imaging modality has a relatively good potential for tumor detection along with tissue penetration with less background scattering (compared to conventional fluorescent probes). Thus, combinations with MRI that can provide deeper anatomical information, arise as a very interesting option [108,109,110,113]. Others trends than the combination of MRI with optical imaging are also developed for other diagnosis applications, mainly combining MRI and positron emission tomography (PET) [115], or MRI and X-ray imaging [116,117].

In more anecdotal literature reports, SPIONs are also used as tools to understand and optimize experimental developments. As examples, SPIONs were used as sensors to optimize bio-targeting strategies e.g., used as sensors to analyze and optimize the effects of surface properties and composition, in a targeted accumulation in artery models [118] or the cell surface [119]. As a last remark, works on SPIONs synthesis are also and still undertaken, to find alternatives to the (classical) thermal decomposition electrochemical reaction [107], or using enzyme mimicking catalytic nanomaterials as new way synthesis of SPIONs (avoiding thermal decomposition and high temperatures) [23,106].

## 6. Conclusions

IONPs, and more precisely magnetite SPIONs, have gained increased interest in past decades in nanomedicine. This is why we chose here to summarize essential information to begin a study of IONPs. Not only can these magnetic NPs possess all assets of NPs dedicated to biomedical uses but they also exhibited additional properties owing to their inherent magnetism, so-called superparamagnetism. It has been overviewed in this work, the main notions about magnetism of NPs and the stringent requirements to fulfill to apply SPIONs into nanomedicine. A focus was on IONPs in order to show why magnetite has become the most used IONP. The impacts of IONP design (surface properties, size, and shape) were all mentioned to bring to light the careful attention that must be paid to prepare IONPs with the desired magnetic and physico-chemical properties. Although magnetite and other SPIONs were described as efficient NPs for applications involving magnetism, it seems that some debate remains concerning the in vivo fate of SPIONs once administered to living beings. Consequently, preclinical research needs to continue in such directions in order to provide a better understanding of the in vivo impact of SPIONs on health and to provide significant data to go further for human applications. We also emphasized the presently reported major routes of synthesis of magnetite with several examples to show the potential of each method and also to highlight the wide range of studies already available and how such kinds of magnetic nanotechnology can be tuned by playing with chemistry and the conditions of preparation.

## Figures and Tables

**Table 1:** Chemical formula and color of main iron oxides (Reproduced with permission from Bruce, I.J. et al., Journal of Magnetism and Magnetic Materials; published by Elsevier B.V., 2004. from ref [33]).

Iron Oxide	Chemical Formula (Current Name)	Color
Ferrous oxide (iron(II) oxides)	FeO (Wüstite)	Black
Mixed-oxide (iron(II, III) oxides)	Fe_3_O_4_ or FeO.Fe_2_O_3_ (Magnetite)	Black
Ferric oxides (iron(III) oxides)	α-Fe_2_O_3_ (Hematite) β-Fe_2_O_3_ γ-Fe_2_O_3_ (Maghemite) ε-Fe_2_O_3_	Grey, brown, red

**Table 2:** Magnetic properties of iron oxides particles and NPs (Reproduced with permission from Teja, A.S. et al., Progress in Crystal Growth and Characterization of Materials; published by Elsevier Ltd., 2009. from ref [5]).

Iron Oxide	Hematite	Maghemite		Magnetite	
Magnetic saturation	0.3 A.m^2^/kg	60–80 A.m^2^/kg		92–100 A.m^2^/kg	
Curie Transition	~1000 K	~820–980 K		~850 K	
Grain size	-	≥10 nm	≤10 nm	≥6 nm	≤6 nm
Magnetism ordering at room temperature	Ferromagnetic	Ferrimagnetic	SPM	Ferromagnetic	SPM
